# Expression of Osteoblast-Specific Factor 2 (OSF-2, Periostin) Is Associated with Drug Resistance in Ovarian Cancer Cell Lines

**DOI:** 10.3390/ijms20163927

**Published:** 2019-08-13

**Authors:** Karolina Sterzyńska, Dominika Kaźmierczak, Andrzej Klejewski, Monika Świerczewska, Karolina Wojtowicz, Marta Nowacka, Jacek Brązert, Michał Nowicki, Radosław Januchowski

**Affiliations:** 1 Department of Histology and Embryology, Poznan University of Medical Sciences, Święcickiego 6 St., 61-781 Poznań, Poland; 2 Department of Nursing, Poznan University of Medical Sciences, Smoluchowskiego 11 St., 60-179 Poznań, Poland; 3 Department of Obstetrics and Women’s Diseases, Poznan University of Medical Sciences, Polna 33 St., 60-535 Poznań, Poland

**Keywords:** Osteoblast-Specific factor 2 (OSF-2), extracellular matrix (ECM), ovarian cancer, drug resistance

## Abstract

One of the main obstacles to the effective treatment of ovarian cancer patients continues to be the drug resistance of cancer cells. Osteoblast-Specific Factor 2 (OSF-2, Periostin) is a secreted extracellular matrix protein (ECM) expressed in fibroblasts during bone and teeth development. Expression of OSF-2 has been also related to the progression and drug resistance of different tumors. The present study investigated the role of OSF-2 by evaluating its expression in the primary serous ovarian cancer cell line, sensitive (W1) and resistant to doxorubicin (DOX) (W1DR) and methotrexate (MTX) (W1MR). The *OSF-2* transcript (real-time PCR analysis), protein expression in cell lysates and cell culture medium (western blot), and expression of the OSF-2 protein in cell lines (immunofluorescence) were investigated in this study. Increased expression of *OSF-2* mRNA was observed in drug-resistant cells and followed by increased protein expression in cell culture media of drug-resistant cell lines. A subpopulation of ALDH1A1-positive cells was noted for W1DR and W1MR cell lines; however, no direct co-expression with OSF-2 was demonstrated. Both drugs induced *OSF-2* expression after a short period of exposure of the drug-sensitive cell line to DOX and MTX. The obtained results indicate that OSF-2 expression might be associated with the development of DOX and MTX resistance in the primary serous W1 ovarian cancer cell line.

## 1. Introduction

Chemoresistance of cancer cells—inherent or developed during treatment—remains an obstacle to the effective treatment of ovarian cancer patients [1]. Hundreds of different molecules are already recognized to be involved in various mechanisms of drug resistance in cancers. In general, these mechanisms are subdivided into cancer-cell-specific ones and cancer-tissue-specific ones, but in most cases, they play together to protect cancer cells against chemotherapy. Among the cancer-cell-specific mechanisms, the most important ones are related to DNA repair, drug inactivation, and drug removal from cancer cells by drug transporters from ABC (ATP—binding cassette) family [2]. However, an increasing body of evidence indicates that tumor-tissue-specific mechanisms can play a leading role in drug resistance in vivo [3,4]. One of the characteristic features of tumor tissues is abundant expression of extracellular matrix (ECM) components, like proteoglycans and collagens, as well as the presence of many soluble factors [3,4]. These features can limit drug delivery to cancer cells by limited diffusion [5] or by the direct binding of anticancer drugs, such as doxorubicin (DOX), paclitaxel (PAC), and methotrexate (MTX) [6]. Additionally, ECM components—both structural, like laminins and collagens, or soluble, like growth factors and matricellular proteins—can interact with cancer cell surface receptors (mainly integrins) and activate different drug resistance mechanisms [3,4]. This kind of resistance, known as cell-adhesion-mediated drug resistance (CAM-DR) is observed both in vivo [7] and in vitro [8], as it was observed in drug-resistant cancer cell lines of breast [9] and ovarian origin [10,11,12,13].

Osteoblast-Specific Factor 2 (OSF-2), also known as a Periostin (POSTN), is a secretory glycoprotein expressed as at least six different isoforms (www.ensembl.org, ENSG00000133110) with a molecular mass from 83.5 to 93.5 kDa [14]. In physiological conditions, it is produced by fibroblasts and secreted to the extracellular matrix [15], whereby interaction with cell receptors, proteases, and other molecules can regulate cell functions [16]. Structurally, the OSF-2 is composed of four main domains: (1) the N-terminus domain, containing a signal peptide; (2) the cysteine-rich EMI domain, responsible for dimerization or oligomerisation [17,18] as well as the binding of ECM proteins, like fibronectin [19] and collagen [18]; (3) the tandem of four fasciclin 1 (FAS1) domains, responsible for interacting with cellular integrins [14,18]; and (4) the C-terminal region (CTR), responsible for proteoglycan [18] and collagen type I and V binding [14]. The EMI domain is responsible for protein dimerization or oligomerization, as the OSF-2 with higher molecular mass was detected [18]. The presence of dimers or oligomers results from disulphide binding [17], although the presence of other dimers was also reported [20,21]. As shown by the structure, the protein has an ability to interact with ECM, which can be important from a CAM-DR point of view. Although physiologically, OSF-2 is expressed in the bones, teeth [22], and heart [23] during development, its expression was also reported in many cancers [14].

In breast cancer, OSF-2 protein expression was mainly detected in the cytoplasm of cancer cells [24,25], but expression was also noted in the nucleus [25] and in the tumor-surrounding tissue [26]. High OSF-2 expression was also observed in lymph node metastasis, where it correlated with the stage of breast cancer [26]. In lung cancer, OSF-2 expression was found in the cytoplasm of tumor epithelia, in stromal cells, and in the ECM [27]. What is more, OSF-2 positive non-small cell lung cancer patients had worst five-year survival [28]. In colorectal carcinoma, the expression was mainly detected in cancer cell areas, with expression statistically increased in metastasis when compared to primary tumors [29]. In serous ovarian cancer patients, OSF-2 expression was significantly higher in metastasis than in primary tumors [30], whilst in another ovarian cancer study, OSF-2 expression was correlated with disease progression and promotion of cancer angiogenesis and metastasis [31].

Periostin expression was also correlated with chemoresistance in two independent ovarian cancer studies. It was identified as one of the reactive stroma genes expressed in cancer-associated fibroblasts (CAFs), with statistically higher expression in chemoresistant, high-grade serosum and endometrioid tumors than in chemosensitive ones. In vitro experiments proved that OSF-2 stimulated the resistance of ovarian cancer cells to carboplatin and PAC [32]. Another study on epithelial ovarian cancer revealed association of strong expression of periostin with a poor prognosis, and platinum resistance and correlation with the FIGO stage (fr. Fédération Internationale de Gynécologie et d’Obstétrique; ang. International Federation of Gynecology and Obsterics). On the other hand, the periostin treatment induced cisplatin (CIS) resistance through serine/threonine protein kinase-AKT-pathway activation in in vitro experiments [33].

To investigate the role of OSF-2 in drug resistance development, we used a model of ovarian cancer, the most lethal gynaecological malignancy [34]. At the beginning of the treatment, ovarian cancer is one of the most well-responding tumors; however, in most cases, it does develop drug resistance [2]. In this study, we used DOX- and MTX-resistant ovarian cancer cell lines. DOX is a cytotoxic drug used in second-line chemotherapy of ovarian cancer [35], and acts as an inhibitor of DNA topoisomerase II, which can also intercalate into DNA and block DNA replication and transcription [36], leading to DNA breakage and cell death. On the other hand, MTX is a folate antagonist that inhibits folate reductase (DHFR), which inhibits DNA synthesis and results in cell proliferation reduction [37]. Among others, resistance to DOX and MTX may be associated with overexpression of drug transporters from the ABC family [38], although the mechanism associated with ECM expression also seems to play an important role [39].

Previously, from the drug-sensitive W1 ovarian cancer cell line, we have developed a series of cell lines resistant to CIS, PAC, DOX, and topotecan (TOP)—drugs used in ovarian cancer chemotherapy, as well as vincristine (VIN) and MTX, which are not used in standard ovarian cancer treatment [40]. These cell lines were further characterized according to ECM protein expression, and among others, elevated levels of OSF-2 in DOX- and MTX-resistant cell lines were detected [39]. In this study, we examined OSF-2 expression at mRNA and the protein levels in DOX- (W1DR) and MTX-resistant (W1MR) cell lines and their corresponding media. We also showed that OSF-2 can be involved in the response to DOX and MTX at the very early stages of treatment.

## 2. Results

### 2.1. OSF-2 Gene Expression in Different Ovarian Cancer Cell Lines

**Figure 1:**
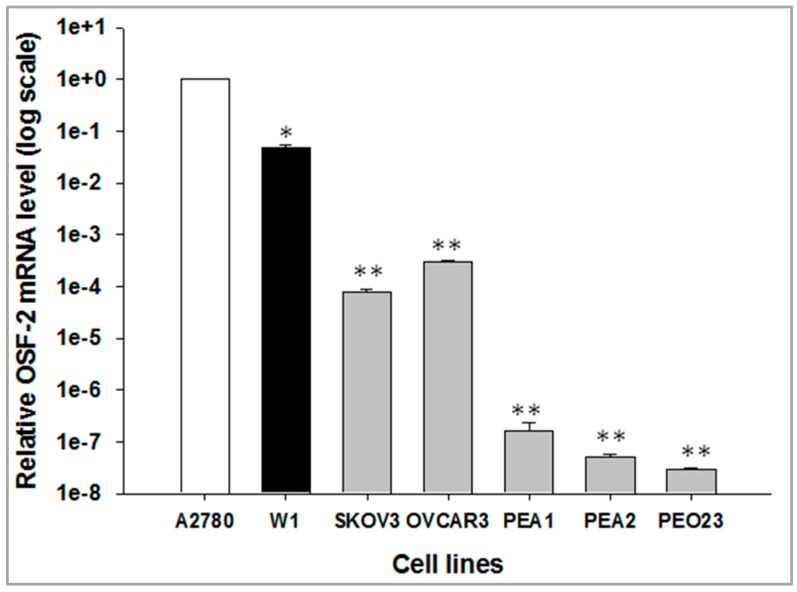
Expression analysis (Q-PCR) of the osteoblast-specific factor 2 (OSF-2) transcript in the different ovarian cancer cell lines. The figure presents the relative gene expression in cell lines (grey bars or black bar for W1) with respect to that in the A2780 cell line (white bar), which was assigned a value of 1. The values were considered significant at * *p* < 0.05 and ** *p* < 0.001, and are presented in log scale.

In the first step of our study, expression of the *OSF-2* gene in the following ovarian cancer cell lines was examined: high-grade serous—OVCAR3, PEA1, PEA2; low-grade serous—PEO23; serous—SKOV-3; endometroid adenocarcinoma - A2780 and primary ovarian cancer cell line—W1. The highest *OSF-2* gene expression was observed in the A2780 cell line, and was assigned as 1. In comparison, for the W1 cell line, about a 20-fold lower expression level was noted. In the consecutive investigated cell lines, expression levels were much lower. Due to big differences in expression level among the examined cell lines, the results are presented as a logarithmic scale (Figure 1).

### 2.2. OSF-2 Gene Expression in Drug-Resistant Ovarian Cancer Cell Lines

**Figure 2:**
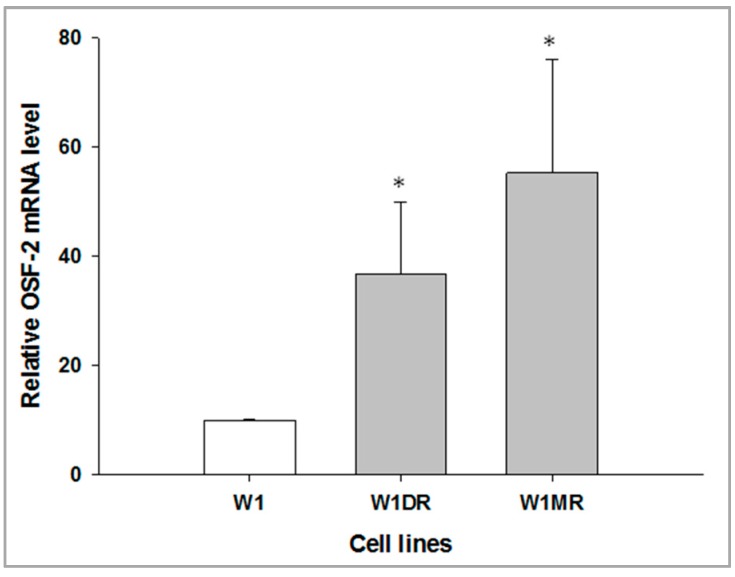
Expression analysis (Q-PCR) of the *OSF-2* transcript. The figure presents the relative *OSF-2* gene expression in DOX- and MTX-resistant cell lines (grey bars) with respect to the W1 drug-sensitive cell line (white bar), which was assigned a value of 1. The values were considered significant at * *p* < 0.05.

In our collection, we possessed a set of drug-resistant cell lines derived from the W1 and A2780 cell lines described previously [40,41]. Microarray analysis indicated that *OSF-2* increased in DOX- and MTX-resistant W1 cell lines [39]. Thus, for more detailed analysis, we used only DOX- and MTX-resistant W1 cell lines. To determine whether the development of drug resistance is associated with the *OSF-2* overexpression, expression of the *OSF-2* mRNA was determined in DOX- and MTX-resistant sublines. We observed statistically significant (*p* < 0.05) increased levels of the *OSF-2* transcript in both cell lines (Figure 2).

### 2.3. OSF-2 Protein Expression in Drug-Resistant Ovarian Cancer Cell Lines

**Figure 3:**
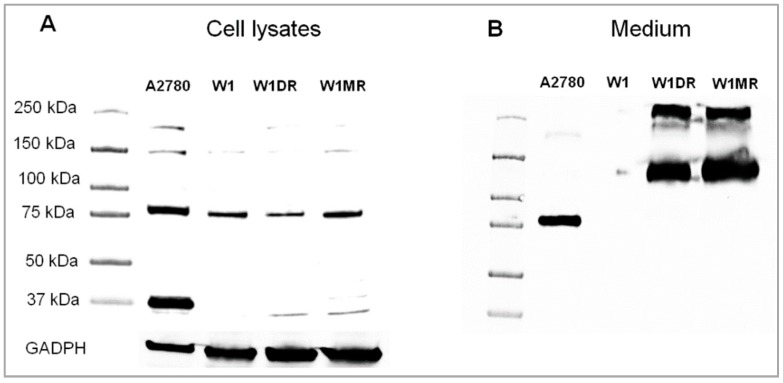
OSF-2 protein expression analysis in: (**A**) cell lines: for A2780—the most intensive bands with masses of 37 and 85 kDa; for W1, W1DR, and W1MR—one prevalent band with a mass of 85 kDa, and (**B**) corresponding media: for A2780—one distinctive band with a mass of 85 kDa; for W1DR and W1MR—highly intensive bands of about 120 kDa and over 250 kDa. The cellular proteins and proteins isolated from the media were separated using 7% PAGE and transferred to a PVDF membrane, which was then immunoblotted with either primary Ab or HRP-conjugated secondary Ab. A primary anti-GADPH Ab was used as a loading control for the cell lysates.

The protein expression analysis was conducted for both cell lines and corresponding media, since OSF-2 is a secretory protein. Additionally, expression of the OSF-2 protein with different molecular mass was described in the literature [17,20,21]. Therefore, we were interested in whether there could be any detectable difference between OSF-2 expression in cell lysates and culture media taken from the investigated cell lines. Western blot analysis conducted on cell lysates revealed the presence of different bands. The highest band intensity was observed in a A2780 cell line that was used as a positive control. The most intensive band corresponded with a mass of 37 and 85 kDa, and the less intensive to 150 kDa and about 200 kDa. In W1 and drug-resistant cell lines, only one prevalent band was observed that corresponded with a mass of about 85 kDa. The intensity of this band was comparable for all cell lines. (Figure 3A). In the next step, we have analyzed the OSF-2 protein expression in cell culture media. In the medium taken from the A2780 cell line, one distinctive band of mass of about 85 kDa was observed. In the medium from the drug-sensitive W1 cell line, no detectable OSF protein was observed, but on the contrary, W1-derived drug-resistant cell lines showed high intensive bands of about 120 kDa and over 250 kDa (Figure 3B).

### 2.4. Early Response to DOX and MTX Treatment in Ovarian Cancer Cell Lines

**Figure 4:**
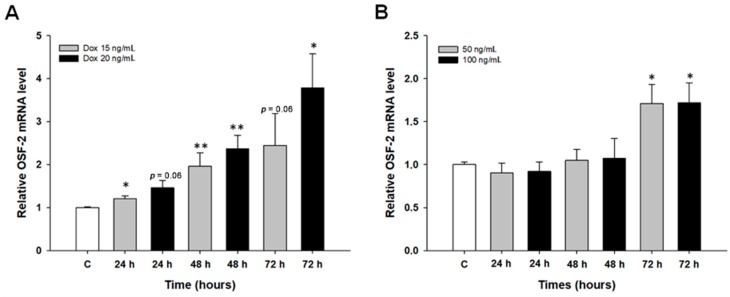
Expression analysis of the *OSF-2* gene in the W1 cell line after a short period of exposure to DOX, with (**A**) a maximum increase in transcript level of about 3.5-fold after 72 h of treatment, and (**B**) a maximum increase in transcript level of about 1.7-fold with MTX after 72 h of treatment. The figure presents relative gene expression in DOX- or PAC-treated cells (grey and black bars) with respect to the untreated control (white bars) assigned as 1. The values were considered significant at * *p* < 0.05, and ** *p* < 0.01.

The second part of our study focused on the early response to DOX and MTX treatment. In these experiments, the W1 drug-sensitive cell line was treated with DOX (15 and 20 ng/mL) and MTX (50 and 100 ng/mL) for 24, 48, and 72 h. Then, changes in gene expression were investigated. The dose- and time-dependent increase in *OSF-2* transcript level was noted (*p* < 0.05 with exception of point 20 ng/mL at 24 h and 15 ng/mL at 72 h) in response to DOX treatment with a maximum increase in transcript level of about 3.5-fold after 72 h of treatment (Figure 4A). A similar result was obtained as an effect of MTX treatment, where a statistically significant increase in *OSF-*2 transcript level after 72 h of treatment (*p* < 0.05) with a maximum increase in transcript level of about 1.7-fold (Figure 4B) was observed.

### 2.5. ALDH1A1 Expression in Drug-Sensitive and Drug-Resistant Cell Lines

**Figure 5:**
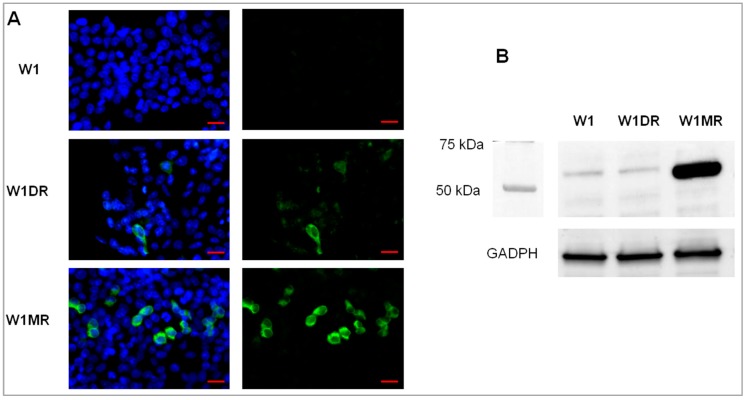
(**A**) Immunofluorescence visualization of ALDH1A1 protein expression in the W1, W1DR, and W1MR cell lines. W1—no ALDH1A1-positive cells; W1DR—a few cells with high expression of ALDH1A1 (less than one percent); W1MR—clear cell subpopulation with high expression of ALDH1A1. ALDH1A1 was detected using the anti-ALDH1A1 antibody and MFP488-conjugated secondary antibody (green). To visualize the cell nuclei, the cells were mounted with a DAPI-containing mounting medium (blue). Objective ×40. Scale bar = 20 µm. (**B**) ALDH1A1 protein expression analysis in the cell lines. The cellular proteins were separated using 7% PAGE and transferred to a PVDF membrane, which was then immunoblotted with either primary Ab or HRP-conjugated secondary Ab. A primary anti-GADPH Ab was used as a loading control for the cell lysates.

Previously, we observed an ALDH1A1 CSCs-like population in PAC- and TOP-resistant ovarian cancer cell lines [13,42]. In the current study, we were interested in whether ALDH1A1-positive cells could also be present and related to DOX and MTX resistance of ovarian cancer cell lines. The immunofluorescence analysis showed that there were no ALDH1A1-positive cells in the W1 drug-sensitive cell line. In the W1DR cell line, we could only observe a few cells with high expression of ALDH1A1 (less than one percent) and some cells with low ALDH1A1 expression. In contrast, a clear cell population with high expression of ALDH1A1 was observed in MTX-resistant cells (Figure 5A). The cellular content of ALDH1A1-positive cells was confirmed by western blot analysis. The high expression of ALDH1A1 in the W1MR cell line and very low signal in W1 and W1DR cell lines was observed (Figure 5B).

### 2.6. Coexpression of ALDH1A1 and OSF-2 in Drug-Resistant Cell Lines

**Figure 6:**
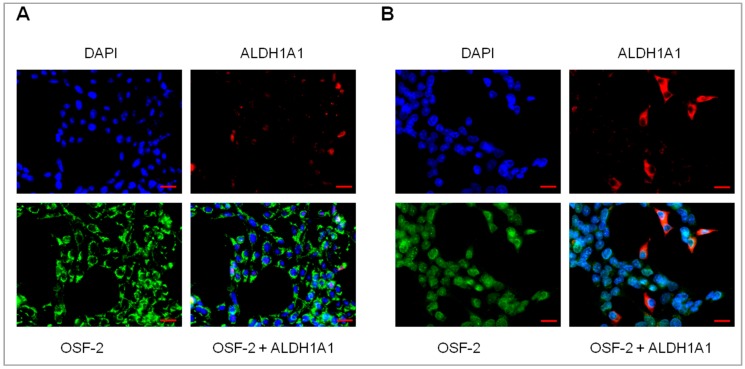
Immunofluorescence visualisation of ALDH1A1 and OSF-2 co-expression in (**A**) the W1DR cell line and (**B**) W1MR cell line. No simultaneous expression of OSF-2 and ALDH1A1 was revealed. ALDH1A1 was detected using the anti-ALDH1A1 antibody and Alexa Fluor^®^594-conjugated secondary antibody (red). OSF-2 was detected using the anti-OSF-2 antibody and Alexa Fluor^®^488-conjugated secondary antibody (green). To visualize the cell nuclei, the cells were mounted with a DAPI-containing mounting medium (blue). Objective x40. Scale bar = 20 µm.

Previously in drug-resistant cell lines, the increased expression of ECM molecules, like COL3A1, COL21A1, and LOX in ALDH1A1-positive cells was observed [42,43]. Therefore, the next step in this study was to check whether there is any relation between OSF-2 and ALDH1A1 expression. Immunofluorescence experiments did not reveal any correlation regarding the simultaneous expression of both proteins, and OSF-2 expression was observed uniformly in all cells (Figure 6A,B).

## 3. Discussion

Chemoresistance of cancer cells still remains one of the obstacles to the effective treatment of ovarian cancer patients The cellular mechanisms of resistance are already well-described in the literature. Cancer-tissue-specific mechanisms are still being investigated and need further evaluation. This is important especially from a clinical point of view, as it was hypothesized that the tumor microenvironment is a dominant force in multidrug resistance and low treatment efficacy of cancers [44]. These mechanisms are strictly related to the tumor microenvironment that is characterized by abundant expression of extracellular matrix components, and among them, OSF-2 [4]. Expression of ECM molecules is not only restricted to cancer-associated fibroblasts (CAFs), but is also observed in cancer cells of epithelial origin [10,12,13,44,45]. In the last decade, expression of different ECM molecules was also described in drug-resistant cancer cell lines of different origin, indicating that ECM-related mechanisms could be involved in drug resistance in vitro [9,10,11,12,13,39,46].

Using the RNA microarray technique, we have analyzed the expression of ECM molecules in ovarian cancer cell lines resistant to CIS, PAC, DOX, TOP, VIN, and MTX. The obtained results found OSF-2 to be overexpressed in cell lines resistant to DOX and MTX [39]. The elevated levels of OSF-2 was already described as being potentially related to the progression of many cancers [24,25,26,27,28,29,30,31] and development of drug resistance, both in vivo and in vitro [32,33]. Therefore, we decided to carry out a detailed analysis of its expression.

The expression analysis conducted for the different ovarian cancer cell lines confirmed the very high expression of *OSF-2* in A2780 endometroid adenocarcinoma [31], whereas it was very low for high-grade and low-grade serous ovarian cancer cell lines. This experiment was followed by expression analysis in drug-resistant OC cell lines. Although the results of our previous study conducted on CIS-, TOP-, and PAC-resistant A2780 cell lines did not reveal any changes in *OSF-2* expression [46], we found increased *OSF-2* transcript levels in DOX- and MTX-resistant W1 cell lines. Since PCR primers used in our study recognize all of the OSF-2 isoforms, a more detailed western blot analysis was performed. As shown in Figure 3, a pattern of isoforms of different sizes was present in drug-sensitive and resistant cell lysates. Since the expression of OSF-2 at the protein level in cell lysates did not fully reflect increased expression at the transcript level, the next step was to investigate its presence in cell culture media. In accordance with the assumptions and on the basis of knowledge about the biological role of OSF-2 in the ECM [16], we confirmed the presence of periostin isoforms in culture media. The pattern differed from that observed for cell lysate analysis (37 and 85 kDa), and proteins of different molecular mass were observed as the most pronounced for W1DR and W1MR drug-resistant cell lines (120–130 kDa and over 250 kDa). Thus, it seems that most of the OSF-2 produced by drug-resistant cell lines was secreted outside the cells into cell culture media. OSF-2 with high molecular mass was also detected by others. Periostin dimers with molecular mass of about 180 kDa were present in mouse connective tissue, and these dimers were probably formed by disulphide bonds because they disappeared in the reduction condition [17,19]. The recombinant periostin monomers and dimers were also observed by Sidhu et al. in an asthma study [47]. In other studies, periostin with a molecular mass of 85 and 170 kDa was shown in different cancer cell lines and corneal fibroblasts [20]. However, in these studies [20,47], the nature of dimers was not investigated. Eventually, the presence of OSF-2 with a molecular mass of 85–95 kDa and 170 kDa was also observed in cell culture medium of periostin-transfected SKOV-3—the ovarian adenocarcinoma cell line, and C848—the primary ovarian cancer cell line [21]. It is worth mentioning that the 170 kDa form was stable under reducing conditions, suggesting that dimerization did not result from disulphide bonds [21]. To check whether the character of the high-molecular-weight protein in our study resulted from the presence of disulphide bonds, the experiments were carried out under reducing and non-reducing conditions (not shown). Since bands of the same molecular weight were observed, we asked the question of where the high molecular mass protein came from. The high mass of this protein may indicate that it represents dimers or oligomers, rather than alternative splicing variants. Because OSF-2 can interact with integrins, especially αvβ3 [21,48] and αvβ5 heterodimers [21] and activates Akt and FAK phosphorylation [48], it is possible that OSF-2 dimers and multimers activate these pathways more effectively, leading to drug resistance in our cell lines. In the ovarian cancer study, the ES-2 cell line growing on OSF-2/fibronectin-coated dishes was much more resistant to PAC and carboplatin than cells growing on plates coated with fibronectin alone, indicating that extracellular periostin can promote drug resistance in vitro [32]. In a breast cancer study, the silencing of periostin expression by siRNA in CSCs isolated from tumor patients led to increased sensitivity to CIS, docetaxel, and epirubicin [49]. In a more detailed study, Sung et al. demonstrated that treatment of the A2780 ovarian cancer cell line with OSF-2 led to the activation of the AKT-signaling pathway and as a result to CIS resistance. Furthermore, the effect of OSF-2 was abolished by the AKT inhibitor MK-2206 [33]. The AKT pathway was also involved in periostin-induced expression of survivin and resistance to oxaliplatin and fluorouracil in colon cancer cell lines SW480 and HT-29 [50]. Since our microarray collection contained data of all our resistant cell lines, we could confront this result as we had analyzed survivin expression in all of them. However, no difference was found in the expression of survivin between drug-sensitive and resistant cell lines, suggesting that in our cell lines, OSF-2 induced drug resistance through another mechanism.

As previously reported, most studies on the effects of cytotoxic drugs describe the difference in gene expression between drug-sensitive and resistant cell lines. The results of our previous experiments indicate that the expression of some genes changes after a short exposure time of cells to cytotoxic drugs [51,52,53]. Importantly, the expression of these genes was further up- and down-regulated in established drug-resistant cell lines. We have also confirmed this observation in our studies for the analysis of *OSF-2* expression after a short period of treatment of a sensitive OC cell line with DOX or MTX. In a similar experiment conducted by Xiao et al., increased expression of periostin in SW480 and HT-29 colon cancer cell lines treated with oxaliplatin and fluorouracil was demonstrated for 24 to 72 h [50]. The results of these experiments may suggest an important role of OSF-2 in the first line of defense against cytotoxic drugs (primary resistance) before other mechanisms of drug resistance appear.

Different models of drug resistance development are described in the literature. In the last 10 years, one of the most-studied models of drug resistance is the one based on the theory of CSCs [54]. Several markers are used to identify CSCs in different cancers, and among them, the aldehyde dehydrogenase isoform 1A1 (ALDH1A1) expression analysis is found [55]. CSCs, described as ALDH1A1-positive populations, were described also in ovarian cancer in vivo [56], and in OC drug-resistant cell lines [57]. Since we previously described the subpopulations of ALDH1A1-positive cells in PAC- and TOP-resistant ovarian cancer cell lines derived from W1 and A2780 cell lines [13,42], we wanted to specify whether such dependence could be observed for W1DR and W1MR cell lines. Indeed, in both resistant cell lines, the variable number of ALDH1A1-positive cells was noted. Previously, we reported that CSCs expressed a higher level of some ECM molecules, like COL3A1, COL21A1, or LOX [13,43]. In a breast cancer study, CSCs isolated from patients’ tumors identified as a CD44+ population expressed significantly higher levels of OSF-2 in comparison to the CD44- population [49]. In contrast to those results, the co-expression study did not confirm any correlation between CSCs and OSF-2 expression, and all cells showed equal OSF-2 levels independently of the ALDH1A1 expression. The different results of both experiments may be due to the following reasons: 1. Different markers were used for CSCs identification; 2. different types of cancers were the origin of the cells; 3. in contrast to the breast cancer study, we used drug-resistant cell lines, that grew in the presence of the cytotoxic drugs. In such conditions, OSF-2 production can be important for the cell to survive. We previously made a similar observation for PAC- and TOP-resistant cell lines, where although only a small population of cells was ALDH1A1-positive, all cells showed multidrug drug-resistant protein expression (P-gp—P-glycoprotein, and BCRP—breast-cancer-resistant protein) [13,42].

OSF-2 expression seems to play a role in drug resistance—not only in vitro, but also in vivo. In two independent ovarian cancer studies, periostin expression in tumor stroma was associated with chemotherapy resistance. In a study by Sung et al., stromal OSF-2 expression was significantly higher in platinum-resistant patients, and correlated with the FIGO stage and tumor recurrence rate after the first treatment. Patients with high OSF-2 expression in stroma also had significantly lower overall survival (OS) and progression free survival (PFS) than those with low expression. In contrast, expression in tumor cells did not correlate with patients’ survival; however, patients with high OSF-2 expression in both compartments had shorter OS and PFS. [33]. In a study by Ryner et al. performed on high-grade serous and endometrioid ovarian tumors, OSF-2 was one of the reactive stroma genes that was upregulated in chemoresistant tumors, in comparision to chemosensitive ones. OSF-2 was restricted to tumor-associated fibroblasts, and was not present in cancer cells [32].

Taken together, periostin seems to play a role in drug resistance, tumor growth, and metastasis in many cancers. As metastatic tumors are usually much more resistant to chemotherapy, we can suppose that periostin could also play a role in the drug resistance of metastatic tumors, such as in the case of drug-resistant cell lines. However, we did not find any data comparing resistant profiles of primary and metastatic tumors together with OSF-2 expression.

## 4. Materials and Methods

### 4.1. Reagents and Antibodies

Culture media (RPMI-1640, MEM, and DMEM), fetal bovine serum, antibiotic-antimycotic solution, L-glutamine, sodium private, DAPI mounting medium, and DOX and MTX were purchased from Sigma (St. Louis, MO, USA). Rabbit polyclonal anti-OSF-2 Ab was obtained from Proteintech (Manchester, UK), rabbit monoclonal anti-ALDH1A1 Ab was purchased from Abcam (Cambridge, UK), mouse monoclonal anti-ALDH1A1 Ab was purchased from ABGENT (San Diego, CA, USA), and rabbit polyclonal anti-GADPH Ab was purchased from Santa Cruz Biotechnology (Santa Cruz, CA, USA). Goat policlonal anti-rabbit horseradish peroxidase (HRP)-conjugated Ab was purchased from Cell Signaling (Danvers, MA, USA). The fluorescent secondary antibodies—Alexa Fluor^®^488 Donkey Anti-Rabbit and Alexa Fluor^®^594 Donkey Anti-mouse IgG—were obtained from Jackson ImmunoResearch Laboratories (Cambridgeshire, UK). Western blot reagents (membranes, gels, and protein markers) were purchased from Biorad (Bio-Rad Laboratories, Hemel Hempstead, UK).

### 4.2. Cell Lines and Cell Culture

The human ovarian carcinoma A2780, SKOV-3, and OVCAR-3 were purchased from ATCC (American Type Culture Collection, Manassas, VA, USA). The human ovarian carcinoma PEA1, PEA2, and PEO23 were purchased from Sigma (St. Louis, MO, USA). The human primary ovarian cancer cell line W1 was established from the tumor tissue of an untreated 54-year-old Caucasian female patient diagnosed for serous ovarian adenocarcinoma (G3, FIGO IIIc). Cells grow as a monolayer, and present an epithelial morphology and adherent growth model. The W1 subline, resistant to MTX (W1MR) and DOX (W1DR) were obtained by exposing W1 cells to the MTX or DOX at incrementally increasing concentrations. The final concentrations used for selecting the resistant cells were 28 ng/mL of MTX, and 100 ng/mL of DOX. The increase in resistance according to the parental drug-sensitive cell line W1 was as follows: 138-fold for W1MR vs. W1, and 10.3-fold for W1DR vs. W1 [40].

Cells were cultured in Minimum Essential Medium Eagle (MEM) medium (A2780), RPMI-1640 medium (W1), RPMI-1640 medium supplemented with 2mM sodium private (OVCAR-3, PEA1, PEA2, PEO23), or Dulbecco’s Modified Eagle Medium (DMEM) (SKOV-3) supplemented with 10% fetal bovine serum, 200 mML-glutamine, penicillin (100 units/mL), streptomycin (100 units/mL), and amphotericin B (25 μg/mL) at 37 °C in an environment of 5% CO_2_.

### 4.3. QPCR Gene Expression Analysis

The total RNA was extracted from cells using a Gene Matrix Universal RNA purification Kit (EURx Ltd., Gdansk, Poland) according to the manufacturer’s protocol. Reverse transcription was performed using the M-MLV reverse transcriptase (Invitrogen by Thermo Fisher Scientific, Waltham, MA, USA) and a thermal cycler (Veriti 96-well Thermal Cycler) as described in the manufacturer’s protocol. The cDNA was amplified from two µg of RNA. Real-time PCR was performed using the 7900 HT Fast Real-Time PCR System (Applied Biosystems, Foster City, CA, USA), Maxima SYBR Green/ROX qPCR Master Mix (Thermo Fisher Scientific, Waltham, MA, USA), and the sequence-specific primers, as indicated in Table 1 (Oligo.pl, Warsaw, Poland). As the negative control, the sample without cDNA was used. The analysis was conducted under the following thermocycling conditions: a hot start (95 °C for 15 min), then 45 cycles of denaturation at 95 °C for 15 sec, annealing at 60 °C for 30 sec, and extension at 72 °C for 30 sec. Target gene expression levels were normalized to that of: glyceraldehyde-3-phosphate dehydrogenase (GADPH), β-actin (ACTB), hypoxanthine-guanine phosphoribosyltransferase 1 (HRPT1), and beta-2-microglobulin (β2M). After amplification was performed to analyze the product melting temperature, the amplification products were checked by Melt Curve analysis and electrophoresis in 3% agarose gel.

The relative quantification (RQ) method was used for gene expression analysis, where RQ estimates the difference at the level of gene expression against the calibrator (RQ of the calibrator = 1). As a calibrator, the drug-sensitive cell lines (A2780 or W1) were used. The standard formula was applied: RQ = 2^−ΔΔ*C*t^ (where ΔΔ*C*t = Δ*C*t of the sample (drug-resistant line) − Δ*C*t of the calibrator (drug-sensitive line)). The graphs were plotted using Sigma Plot.

### 4.4. Western Blot Analysis

Cell lysates (from 1 × 10^6^ cells/25 μL) were prepared using a RIPA buffer containing a protease inhibitor cocktail (Roche Diagnostics GmbH, Mannheim, Germany), and then, supernatant was collected after centrifugation at 4 °C and 12,000× *g* for 15 min. A Bradford protein assay system (Bio-Rad Laboratories, Hemel Hempstead, UK) was used to determine the protein concentrations. The isolation of proteins from culture media was prepared after a 72 h culture of cells in serum-free media. Next, the media was centrifuged at 15,000 rpm for 30 min at RT, and supernatants were placed in Amicon Ultra-15 3K centrifuge filter devices (Sigma, St. Louis, MO, USA) and centrifuged using a swinging-bucket rotor for 60 min at 4,000× *g*/RT.

For the western blot analysis, 10 μg protein from each sample were resuspended in 4× loading buffer (Bio-Rad Laboratories, Hemel Hempstead, UK) and loaded into each well of a 4–20% mini-PROTEAN^®^ TGX™ precast gel using the SDS-PAGE technique. The proteins were then transferred to a nitrocellulose membrane that was incubated in a blocking solution (5% milk in 0.1 M Tris-HCl, 0.15 M NaCl, 0.1% Tween 20). Next, the membrane was incubated with rabbit policlonal anti-OSF-2 or rabbit monoclonal anti-ALDH1A1 antibodies at dilutions of 1:500, followed by incubation with the appropriate HRP-conjugated secondary antibody. The bands detection was analyzed using a chemical luminescence kit (ECL, Femto Super Signal Reagent) and Hyperfilm ECL (GE Healthcare, Buckinghamshire, UK). The quality of protein loading was made by reblotting the membrane with a rabbit anti-GAPDH antibody (1:1000) and goat anti-rabbit HRP-conjugated antibody (both from Santa Cruz Biotechnology, Santa Cruz, CA, USA)

### 4.5. Immunofluorescence Analysis

For immunofluorescence and double immunofluorescence analysis, cells were cultured in 24-well chamber glass slides and grown to a near-confluent state. The standard procedure was performed as follows. The culture medium was replaced by PBS for rinsing, and next by ice-cold acetone/methanol (1:1) for 10 min for fixation and permeabilization. Then, cells were washed with PBS and blocked in 3% BSA for 30 min at room temperature. After that, the incubation with primary antibodies was prepared. For classic immunofluorescence one type of primary antibody at a time was used (rabbit monoclonal anti-ALDH1A1 antibody, 1:100, Abcam, Cambridge, UK). For double immunofluorescence purpose the cells were incubated with the mixture of two primary antibodies simultaneously: OSF-2 (rabbit polyclonal anti-OSF antibody, 1:200, Proteintech, Manchester, UK) + ALDH1A1 (mouse monoclonal anti-ALDH1A1 antibody. 1:100, ABGENT, San Diego, CA, USA). After 2 h incubation at room temperature, cells were washed with PBS and incubated with secondary antibodies. For single immunofluorescence, the incubation with green dye-labelled Alexa Fluor^®^488 secondary antibody (Donkey Anti-Rabbit IgG, Jackson ImmunoResearch Laboratories, Cambridgeshire, UK) for 1 h at room temperature was proceed. In the double immunofluorescence, the cells were incubated with the mixture of two respective green dye-labelled (Alexa Fluor^®^488, donkey anti-rabbit IgG, 1:400 Jackson ImmunoResearch Laboratories) and red dye-labelled (Alexa Fluor^®^594, donkey anti-mouse IgG, 1:400, Jackson ImmunoResearch Laboratories) secondary antibodies for 1 h at room temperature. Finally, cells were washed with PBS and mounted in DAPI mounting medium. The expression analysis and pictures were taken under fluorescence microscope (Zeiss Axio-Imager.Z1, Carl Zeiss Microscopy GmbH, Oberkochen, Germany).

### 4.6. DOX and MTX Response in Time-Course Experiment

In the time-course experiments, the expression of *OSF-2* was measured after a short period of exposure to a citotoxic agent. The drug-sensitive cell line (W1) was cultured in 1 mL of medium at a density of 0.5 × 10^6^ cells per well (six-well plate). Then, the DOX at 15 and 20 ng/mL concentrations or MTX at 50 ng/mL and 100 ng/mL concentrations were added, respectively (detailed procedure described previously [13]). The incubation proceeded for 24, 48, and 72 h and was followed by cell harvesting, RNA isolation, and gene expression analysis.

### 4.7. Statistical Analysis

Data are presented as standard error of the mean (SEM) and were analyzed using Student’s *t*-test. *p* < 0.05 was considered to indicate a statistically significant difference.

## 5. Conclusions

To our knowledge, this study is the first to describe the expression analysis of OSF-2 in drug-resistant ovarian cancer cell lines. Our research showed elevated levels of OSF-2 expression in W1 DOX- and MTX-resistant ovarian cancer cell lines and corresponding media. The short exposure time of cells to DOX- or MTX-induced expression of OSF-2 in the drug-sensitive cell line, which may suggest its potential role in the primary resistance of this cell line before other mechanisms of drug resistance appear. An extracellularly secreted OSF-2 protein could be involved in CAM-DR. Thus, we have found that the expression of OSF-2 might be associated with the development of DOX and MTX resistance of the primary serous W1 ovarian cancer cell line.

## Figures and Tables

**Table 1:** Oligonucleotide sequences used for RQ-PCR analysis.

Transcript	Sequence (5’-3’ Direction)	ENST Number (Available online: http://www.ensembl.org)	Product Size (bp)
OSF-2	TTCTGACGCCTCAAAACTGA CATTCACGTTGCTCTCCAAA	00000379742	128 bp
GADPH	GAAGGTGAAGGTCGGAGTCA GACAAGCTTCCCGTTCTCAG	00000229239	199 bp
β-actin	TCTGGCACCACACCTTCTAC GATAGCACAGCCTGGATAGC	00000331789	169 bp
HRPT1	CTGAGGATTTGGAAAGGGTG AATCCAGCAGGTCAGCAAAG	00000298556	156 bp
β2M	CGCTACTCTCTCTTTCTGGC ATGTCGGATGGATGAAACCC	00000558401	133 bp
